# Mapping Driver Mutations to Histopathological Subtypes in Papillary Thyroid Carcinoma: Applying a Deep Convolutional Neural Network

**DOI:** 10.3390/jcm8101675

**Published:** 2019-10-14

**Authors:** Peiling Tsou, Chang-Jiun Wu

**Affiliations:** Department of Genomic Medicine, University of Texas, MD Anderson Cancer Center, 1901 East Road, 3SCR5.4101, Houston, TX 77054, USA; peiling.tsou@gmail.com

**Keywords:** Papillary thyroid carcinoma, histopathology, *BRAF^V600E^*, *RAS*, deep learning, convolutional neural network

## Abstract

Papillary thyroid carcinoma (PTC) is the most common subtype of thyroid cancers and informative biomarkers are critical for risk stratification and treatment guidance. About half of PTCs harbor *BRAF^V600E^* and 10%–15% have *RAS* mutations. In the current study, we trained a deep learning convolutional neural network (CNN) model (Google Inception v3) on histopathology images obtained from The Cancer Genome Atlas (TCGA) to classify PTCs into *BRAF^V600E^* or *RAS* mutations. We aimed to answer whether CNNs can predict driver gene mutations using images as the only input. The performance of our method is comparable to that of recent publications of other cancer types using TCGA tumor slides with area under the curve (AUC) of 0.878–0.951. Our model was tested on separate tissue samples from the same cohort. On the independent testing subset, the accuracy rate using the cutoff of truth rate 0.8 was 95.2% for *BRAF* and *RAS* mutation class prediction. Moreover, we showed that the image-based classification correlates well with mRNA-derived expression pattern (Spearman correlation, rho = 0.63, *p* = 0.002 on validation data and rho = 0.79, *p* = 2 × 10^−5^ on final testing data). The current study demonstrates the potential of deep learning approaches for histopathologically classifying cancer based on driver mutations. This information could be of value assisting clinical decisions involving PTCs.

## 1. Introduction

Thyroid cancer is the most common form of endocrine malignancy. The incidence of thyroid cancer has increased 3-fold over the past 30 years [1], and it is estimated by the National Institutes of Health that in 2018 more than 53,000 new cases of thyroid cancer and more than 2000 deaths from these diseases occurred in the United States. Papillary thyroid carcinoma (PTC) is the most common subtype, comprising approximately 80% of thyroid cancers. Current treatment includes surgery, thyroid hormone, and radioactive iodine (RAI). More informative biomarkers, whether molecular or morphological, are critical in the clinical decision-making of PTC [2]. Moreover, since the prognosis of PTCs is generally good with a 5-year survival rate of over 95% [3], it is important to spare patients from overtreatment by distinguishing between indolent and clinically aggressive tumors. For example, a recent publication reported that assessment of *BRAF^V600E^* assist risk stratification of solitary intrathyroid PTC with size between 1 to 4 cm [4], and for thyroid microcarcinoma, it has also been shown that *BRAF* analysis might help to identify tumors that have negligible clinical risk [5]. On the other hand, for patients who develop advanced disease, such as metastatic RAI-refractory disease, treatment options are limited. Targeted therapy might provide some possibilities [6,7,8] and undoubtedly, the identification of relevant mutations is the key.

PTC is a MAPK-driven cancer that has two mutually exclusive drivers: *BRAF^V600E^* and mutated *RAS* with distinct downstream signaling events [9]. The *BRAF^V600E^* mutation is the most common genetic alteration in PTC, occurring in approximately half of all patients and *RAS* point mutations, which includes several specific sites (codons 12, 13, and 61) of N-RAS, H-RAS, or K-RAS genes, were found in 10% to 15% of PTC [10]. Tumors driven by *BRAF^V600E^* do not respond to the negative feedback from ERK to RAF, resulting in high MAPK-signaling [11]. In contrast, tumors driven by RAS signal via RAF dimers that respond to ERK feedback result in lower MAPK-signaling. This differential signaling results in profound phenotypic differences. Importantly, the presence of *BRAF^V600E^* not only correlates with aggressive tumor behaviors [12] but also is associated with decreased ability of tumors to take up RAI [13], leading to a worse prognosis in population studies [14,15].

It has been shown that there exists a strong association between *BRAF^V600E^* and *RAS* mutation status and histopathology. Tumors harboring *BRAF^V600E^* mutation characterize classical papillary pattern and tall cell variation while tumors with *RAS* mutations characterize follicular variant, which possesses the follicular growth pattern and architecture typical of follicular tumors [9]. Correlating morphological patterns with molecular characters using deep learning demonstrates substantial promise [9,16,17]. Given the phenotypic messages present in histopathology usually reflect the collective effects of molecular processes and cellular behaviors, it is possible that *BRAF^V600E^* and *RAS* mutations can be distinguishable through histopathological presentations. To test the hypothesis, in the current proof-of-concept study we trained a deep learning convolutional neural network (CNN) model (Google Inception v3) on histopathology images obtained from The Cancer Genome Atlas (TCGA) to classify PTCs into *BRAF^V600E^* or *RAS* mutations.

## 2. Results

### 2.1. A CNN Framework for Differentiating Mutations of PTC from Histological Images

**Figure 1:**
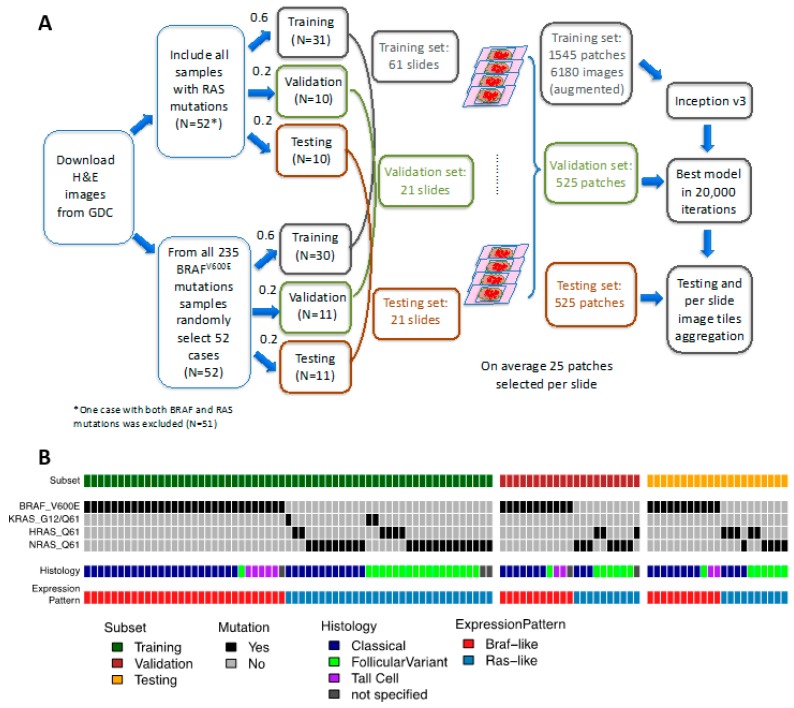
Study cohort. (**A**) Sample selection, allocation, and analysis strategy. Formalin-fixed, paraffin-embedded (FFPE) slides Hematoxylin and eosin (H&E) stained images were downloaded from GDC repository. Samples with *RAS* or *BRAF^V600E^* mutations were allocated into training, validation, and testing sets with ratios of 0.6, 0.2, and 0.2, respectively. On average there were 25 patches selected per slide. The Inception V3 framework was applied on the training set for 20,000 iterations. The model with the highest accuracy in validation set was chosen as the final model. Classifications were run on an independent testing set and the results were aggregated per slide. (**B**) The oncoprint depicting the molecular characteristics and histological subtypes of samples used in this study.

**Figure 2:**
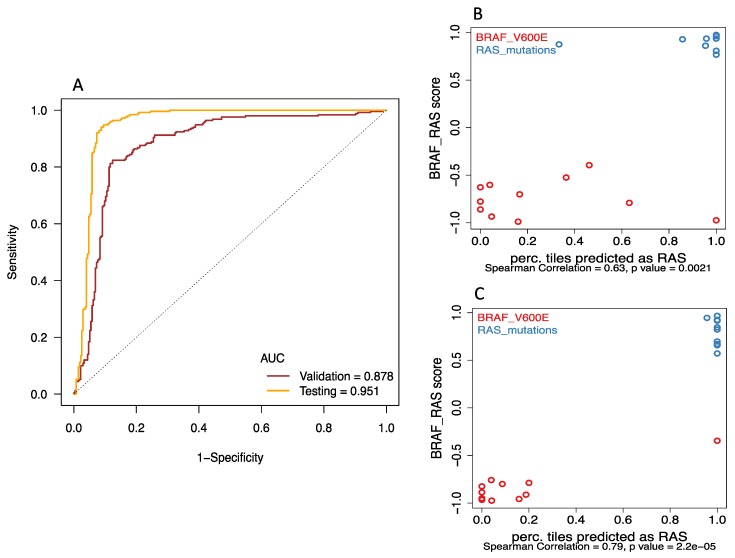
Deep-learning models successfully discriminated *BRAF^V600E^* and *RAS* mutation of papillary thyroid carcinomas from histological images. (**A**) Receiver Operating Characteristic (ROC) curves showed that area under curve (AUC) values for the best model were 0.878 for validation and 0.951 for final testing subsets. (B&C) The correlations between the probability of accurate prediction per tile image and BRS (BRAF-RAS score) were significant in both (**B**) validation (Spearman correlation rho = 0.63, *p* = 0.002) and (**C**) final testing subsets (Spearman correlation rho = 0.79, *p* < 0.001).

The goal of this study was to develop a deep-learning model to accurately classify the most common two mutations in PTCs: *BRAF^V600E^* and *RAS* based on Hematoxylin and eosin (H&E) tissue slide images, which were publicly available from TCGA portal. The sample selection, allocation, and analysis strategies were depicted in Figure 1A. We first examined these two mutations in this cohort and 235 and 52 samples with *BRAF^V600E^* and *RAS* mutations, respectively, were identified. One sample (TCGA-EM-A4FV) was detected to have both mutations and was excluded. Additionally, as shown in Figure 1B, we also categorized histopathological subgroups and mRNA expression patterns of these two groups to further determine whether the results of our prediction would correlate with traditional pathological classification or mRNA expression. As shown in Supplementary Table S1A, classical PTCs dominated the pathological category of *BRAF^V600E^* cases (assigned classical: 76.5%, follicular variant:5.1%, and tall variant:11.5%). For *RAS* mutations, there were 49 samples where pathological reviews were still available. Out of these samples, 29 were assigned as FCV and 20 were assigned as classical PTC, respectively. To compile the list for model construction, all 51 samples with *RAS* mutation were included and 52 samples with *BRAF^V600E^* were selected randomly (Figure 1A). We then split the cohort into training (60%), validation (20%), and final testing (20%) sets. The network was trained, validated, and finally tested using 512 × 512-pixel tiles obtained from nonoverlapping patches. The sampling and image tile augmentation procedures are described in detail in the Methods section. The final model with the best accuracy in validation subset was obtained on iteration 195,000 (Supplementary Figure S1). This model resulted in an AUC of 0.878 on a validation dataset and 0.951 on a final testing dataset (Figure 2A).

### 2.2. Deep-Learning Models Successfully Discriminate of BRAF^V600E^ and RAS Mutation of Papillary Thyroid Carcinomas from Histological Images

Receiver Operating Characteristic (ROC) curve analysis (Figure 2A) showed that *BRAF^V600E^* and *RAS* mutation are predictable using our deep-learning approach. AUC values for the best model were 0.878 for validation and 0.951 for final testing subsets. We included the prediction of an image only if the probability of either *BRAF* or *RAS* class was at least 80%; and reported the prediction of a tumor only if at least 80% image tiles derived from the slide agreed with it. With the 80% exclusion rule, 76% of validation tumors and 95% of final testing tumors had correct predictions, while 5% of validation and 5% of testing tumors had incorrect calls, and 19% of validation tumors could not be predicted. We also evaluated the performance if all tumors and images were used without any exclusion, the accuracy increased to 85% in validation data and remained at 95% in the final testing data.

Interestingly, it seemed that the prediction performed better for predicting *RAS* than for *BRAF*. For the validation subset, the accuracy rate of successful prediction using the cutoff of true rate 0.8 was 63.6% and 90% for the *BRAF* group and *RAS* group, respectively. In the final testing subset, the accuracy of prediction using the cutoff of true rate 0.8 was 90.9% for the *BRAF* group and 100% for the *RAS* group. The confusion matrices in Supplementary Table S2A detail the discrepancies between the different classifications.

**Figure 3:**
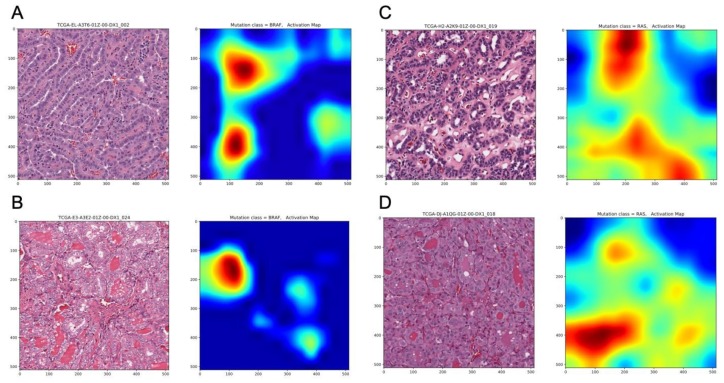
Representative histopathological images with corresponding activation maps of *BRAF^V600E^* and *RAS* mutations. (**A**) *BRAF^V600E^* mutated, Classical Histological Type, (**B**) *BRAF^V600E^* mutated, Follicular Variant Type, (**C**) *RAS* mutated, Classical Histological Type, (**D**) *RAS* mutated, Follicular Variant Type.

In addition, the image-based classification seemed to correlate well with mRNA-derived expression pattern. The correlations between the probability of accurate prediction per tile image and BRS (BRAF-RAS score) were significant in both validation (Figure 2B: Spearman correlation rho = 0.63, *p* = 0.002) and final testing subsets (Figure 2C: Spearman correlation rho = 0.79, *p* < 0.001). Representative histopathological images with activation maps of *BRAF^V600E^* and *RAS* mutation are depicted in Figure 3.

### 2.3. BRAF Mutations Other Than BRAF^V600E^ Were Classified as RAS-Mutated while BRAF Fusions Were Classified as BRAF^V600E^ Group, Respectively

**Figure 4:**
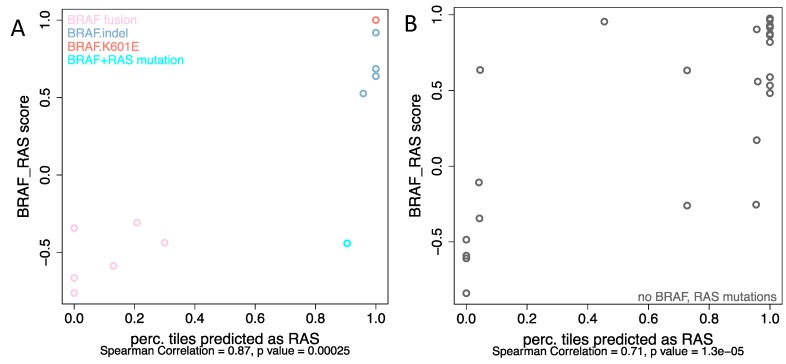
The correlation between BRS score and the possibility of accurate prediction per tile image for (**A**) non-V600E *BRAF* mutations as well as *BRAF*-fusions, (**B**) samples without known *BRAF* or *RAS* alterations.

Five tumors with *BRAF* mutations other than *BRAF^V600E^* were predicted as being from the *RAS* mutation group by our classifier. Consistently, these tumors exhibited *RAS*-like behavior according to BRS score, which were derived from mRNA expression profiles (Figure 4A). This is in line with previous observations that *BRAF ^K601E^* occurs in follicular variant tumors that are mostly *RAS*-like PTCs [18]. On the other hand, the *BRAF* fusions were predicted as *BRAF^V600E^*. Three of the six *BRAF* fusions, including fusion with *MACF1*, *FAM114A2*, and *MKRN1*, were 100% predicted as *BRAF^V600E^* and *SND1_BRAF*, *AP3B1_BRAF*, and *AGK_BRAF* were predicted as *BRAF^V600E^* with the probability of 87%, 79.2%, and 70%, respectively. These 6 cases of *BRAF* fusion were also categorized as *BRAF*-like according to the BRS score. The confusion matrices in Supplementary Table S2B detail the discrepancies between the different classifications.

### 2.4. Analyses from Histological Images Correlate with Molecular Expression for Tumors without BRAF^V600E^ and RAS Mutations

Although the current study mainly focused on differentiating *BRAF^v600E^* and *RAS* mutation, we were wondering how cases without either of these two mutations would be classified. Among these samples, mRNA expression profiles were available in 101 cases with 38 were *BRAF*-like and 63 were *RAS*-like according to the BRS score. Thirty cases (8 *BRAF*-like and 22 *RAS*-like) were randomly selected and tested with the best-performing model. Interestingly, as shown in Figure 4B, even though there were no mutations of either *BRAF* or *RAS* per se, mRNA-based BRS scores correlated well with the information derived from histopathology: 6 of 8 *BRAF*-like tumors (BRS < 0) were assigned to the *BRAF^v600E^* group and 19 of 22 *RAS*-like tumors (BRS > 0) were classified as *RAS* mutated. The confusion matrices in Supplementary Table S2C detail the discrepancies between the different classifications.

## 3. Discussion

The ability to promptly and accurately predict the gene mutations from histopathology images could be of great value regarding risk stratification and treatment guidance for cancer patients. Previous work has shown associations between clinically important mutations and specific patterns of lung adenocarcinoma [17]. Also, Chiang et al. [19] recently demonstrated the relationship between a defining mutation and the unique morphology of a breast cancer subtype. While phenotypic associations of somatic mutations are not routinely used for predicting oncogenic drivers in PTC, this study reported promising predicative capabilities of a deep convolutional neural network, which could potentially help classify *BRAF^V600E^* and *RAS* mutations in PTCs from histopathology images. The performance of our method is comparable to that of recent publications of other cancer types (lung, breast, bladder) using TCGA tumor slides [20] with an AUC of 0.878 for validation and 0.951 for final testing subsets.

Although the classifier was trained with the input of mutation-stratified images, intriguingly, the derived image-based classification correlated well with mRNA-derived expression pattern (BRS scores) as well. One plausible explanation is that these driver mutations dictate the downstream signaling cascades: both mRNA expression profiles and histopathological architectures simply reflect the integrative pictures of these molecular events. Interestingly, it seemed that the prediction of our CNN models performed better for classifying *RAS* than for *BRAF*. This is consistent with the findings from the recent TCGA landmark paper of PTCs [9]. The authors concluded that RL-PTCs and BVL-PTCs are fundamentally different in their genomic, epigenomic, and proteomic profile. Importantly, they also suggested that *BRAF^V600E^* PTC should not be considered a homogeneous group in clinical studies [9].

This study has important limitations. First, this was not based on whole-slide automated image segmentation, and the selection of ROIs within each slide required expert guidance. Future studies will explore more advanced methods for automatic selection of regions and for incorporating a higher proportion of each slide in training and prediction. Moreover, as a proof of concept, we only focused on the two major mutations of PTCs: *BRAF^V600E^* and *RAS*, which estimated to account for around two thirds of the PTC cases. The value of clinical application is somewhat limited without a multi-classifier. Further efforts on genomic alterations other than these two mutations are needed for a more comprehensive mapping between the intriguing connection between molecular and morphological events. Last but not least, in future study the concept of intra-tumoral heterogeneity might need to be taken into consideration. Despite these caveats, we believed that it would still be important to demonstrate that deep convolutional neural networks can potentially be used to assist in molecular pathology and provide useful clinical insights.

## 4. Methods

### 4.1. Sample Selection and Image Processing

To test whether CNNs can be trained to predict gene mutations using images as the only input, whole slide image, genomics and clinical data for matched patient samples were downloaded from the TCGA website (https://gdc.cancer.gov/). The images were downloaded in the native image format, Aperio SVS files, in which they had been scanned. An SVS file stores an image in multiple resolutions, including the highest resolution the image data was captured; for example, in an image that is acquired at a 40× magnification, each pixel is 0.25 × 0.25 microns.

There were 235 and 52 samples with *BRAF^V600E^* and *RAS* mutations, respectively. One sample (TCGA-EM-A4FV) was detected to have both mutations and was excluded. The distribution of histological classification of all samples with *BRAF^V600E^* and *RAS* were listed in Supplementary Table S1A. All 51 samples with *RAS* mutation were included. On the other hand, 52 samples with were selected randomly from 234 cases with *BRAF^V600E^* mutation (Figure 1A). Patient demographic characteristics of the study cohort were listed in Supplementary Table S1B. Patients were randomly assigned to nonoverlapping training (60%), validation (20%), and final testing (20%) sets that were used to train models and evaluate their performance (Figure 1A). Although there was more than one slide for each patient, we chose only one diagnostic, H&E-stained, formalin-fixed, paraffin- embedded (FFPE) sections for our analysis. All selected slides were reviewed and representative regions containing primarily tumor nuclei were manually identified for each slide to avoid images containing tissue-processing artifacts, including bubbles, tissue folds, and chromatic aberration due to inadequate staining. A single 2048 × 2048-pixel svs image was cropped and re-sized to down to 512 × 512-pixel jpg images (HPF: resolution 0.5 μm/pixel). On average, there were 25 patch images per slide. In the end, there were 1525 patches for training, 525 for validation, and also 525 patch tiles for final testing. 

### 4.2. Network Architecture and Training Procedures

Our imaging classification model was based on modified inception v3 architecture. Inception v3 is a deep convolution neural network model, consisting of multiple convolution layers, filter layers, pooling layers, activation layers, one fully connected layer, and a final softmax output layer. The model was modified to allow two-label output classification (DeepPath, https://github.com/ncoudray/DeepPATH).

For *BRAF^V600E^* vs *RAS* mutation classification, we trained the entire network model preloaded with imagenet weights using high-power field (HPF) images in the training set. RMSprop optimizer with initial learning rate 0.1 and a decay factor of 0.16 was used. We implemented an image augmentation approach to convert each patch in the training set into four instances: original, 180° rotation, horizontal, and vertical flips. A total of 6180 training images were used in mini-batches of size 64.

The training jobs were run for 200,000 iterations. We evaluated its classification performance on validation dataset every 5000 iterations. As shown in Supplementary Figure S1, the model with the best accuracy performance in the validation data during the first 200,000 iteration was chosen as the final model. The final testing dataset was used to independently evaluate the non-biased performance.

### 4.3. Validation and Testing Procedures

The final softmax layer of our CNN model calculates the probability of *BRAF^V600E^* or *RAS* mutations of every input image tile. We excluded image predictions if neither the probabilities of *BRAF^V600E^* nor *RAS* mutations were greater than 80%. For every tumor tested, a percRAS score was defined as the percentage of images predicted as *RAS* mutation class. We predicted a tumor as *RAS* mutated if the percRAS score was greater than 0.8; and predicted a tumor as *BRAF* mutated if the percRAS score was less than 0.2. The mutation class would not be predicted if the percRAS score was between 0.2 and 0.8.

### 4.4. Performance Evaluation

#### Metrics for Performance Evaluation of Algorithms

The performance of our CNN model was evaluated by the receiver operating characteristic (ROC) curves. Higher area under the ROC curve (AUC) represents better prediction performance. We also used confusion matrices to summarize the comparison between real mutation statuses and the predicted *BRAF^V600E^*/*RAS* classes. The significance *p* value of the prediction accuracy was estimated by the Fisher’s exact test.

### 4.5. Hardware and Software

Prediction models were trained using TensorFlow (v1.3.0) on a server equipped with Intel(R) Core(TM) i7-7700 CPU@3.60 GHz CPUs, 48 GB RAM, and a NVIDIA GeForce GTX 1070, 8GB RAM graphics card. Image data were extracted from Aperio svs whole-slide image formats. Data processing, model training, and evaluation was done in a Python 3.6 environment.

### 4.6. BRS Scores

BRS scores were used to quantify the extent to which the gene expression profile of a given tumor resembles either the *BRAF^V600E^* or *RAS* mutant profiles as *BRAF*-like (BVL) or *RAS*-like (RL) and obtained from the landmark research of TCGA [9]. Briefly, 391 samples with both exome and RNA sequencing data were compared to derive a 71- gene signature. Correlations with this signature were used to derive a continuous measure (−1 to +1), with BVL- PTCs being negative and *RAS*-like (RL) PTCs. This signature showed strong separation of the *BRAF^V600E^* and *RAS* mutant tumors.

### 4.7. Prediction on Additional PTC Tumor Images

We applied our final model on tissue images from six tumors with *BRAF* fusions, five tumors with non-V600E *BRAF* mutations, one tumor with double *BRAF^V600E^* and *KRAS* mutations, and 30 tumors without *BRAF* or *RAS* mutations. Spearman correlation between tumor BRS scores and prediction percRAS values were estimated. We also used a confusion matrix to compare the predicted classes with the expression pattern (*BRAF*-like or *RAS*-like) of the tumors.

### 4.8. Ethical Statement

The slide images and the corresponding cancer information were de-identified and retrieved from the Genomic Data Commons portal (https://gdc-portal.nci.nih.gov) public repositories and are in whole or part based upon data generated by the TCGA Research Network (http://cancergenome.nih.gov/). The data used were publicly available without restriction, authentication, or authorization necessary.

## 5. Conclusions

To summarize, the current study demonstrates the potential of deep learning approaches for histopathologically classifying cancer based on driver mutations. This information could be of value in assisting clinical decisions involving PTCs.

## Supplementary Materials

The following are available online at https://www.mdpi.com/2077-0383/8/10/1675/s1. Table S1: Demographic characteristics and histological types of the PTC data, Table S2: Confusion matrices of prediction results, Figure S1: Error rates and AUC of models on training, validation, and testing data during the first 20,000 iterations.
