# Supplementary material for: Mapping Driver Mutations to Histopathological Subtypes in Papillary Thyroid Carcinoma: Applying a Deep Convolutional Neural Network
Source: J Clin Med. 2019 Oct 14;8(10):1675. doi: 10.3390/jcm8101675 (PMC6832421; doi:10.3390/jcm8101675)
Supplement: Supplementary file 1 [file jcm-08-01675-s001.zip › SuppFigure_S1.pdf]

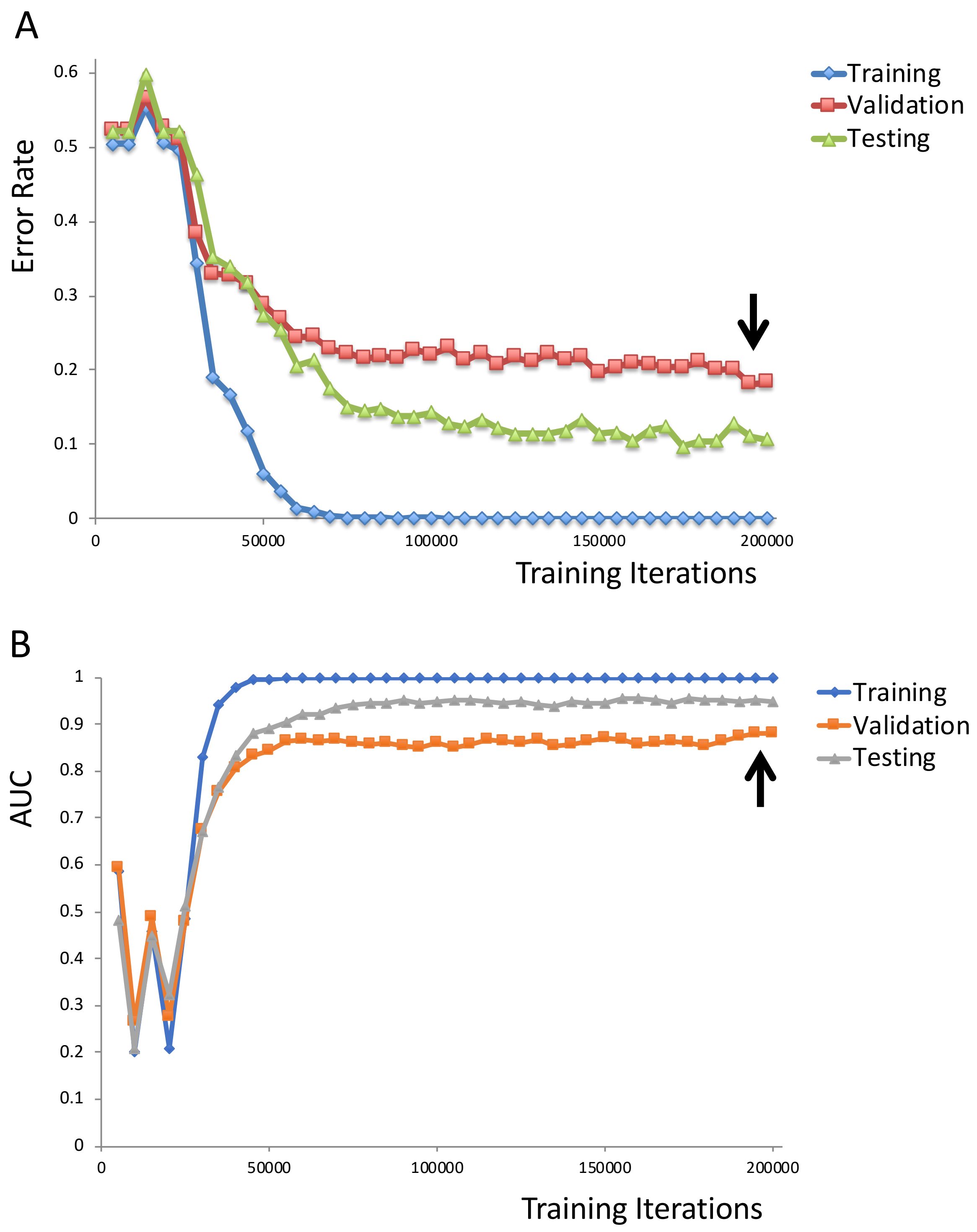

Supplementary Figure S1. (A) Error rates and (B) AUC of models on training, validation, and testing data during the first 20,000 iterations.
Arrow: Model at the 195,000th iteration had the lowest error rate in validation data.
